# Interpretable multitask deep learning model for detecting and analyzing severity of rice bacterial leaf blight

**DOI:** 10.1038/s41598-025-12276-0

**Published:** 2025-07-27

**Authors:** Sudhesh K. M, Aarthi R., Sainamole Kurian. P, Sikha O.K

**Affiliations:** 1 Department of Computer Science and Engineering, Amrita School of Computing, Coimbatore, Amrita Vishwa Vidyapeetham, India; 2 Department of Plant Pathology, College of Agriculture, Kerala Agricultural University, Padannakkad, Kerala India; 3 Department of Engineering, BCN Medtech, Universitat Pompeu Fabra, Barcelona, Spain

**Keywords:** Disease Progression Analysis, BLBVisionDB, Artificial Inoculation, Rice Bacterial Leaf Blight (BLB), Convolutional Block Attention Module (CBAM), Grad-CAM visualizations, Computer science, Scientific data

## Abstract

Rice Bacterial Leaf Blight (BLB), caused by *Xanthomonas oryzae* pv. *oryzae* (Xoo), is a major threat to rice production due to its rapid spread and widespread impact. Early detection and stage-specific classification of BLB are essential for timely intervention, particularly in complex environments with cluttered backgrounds and overlapping symptoms. This study introduces RCAMNet, a novel multi-task framework designed for accurate classification and severity analysis of BLB. The proposed approach begins by generating multiclass segmentation masks using three candidate methods: MultiClass U-Net, DeepLabv3, and Detectron2 (used here for its instance segmentation capability). In the second phase, a dual-path attention mechanism is employed. The Convolutional Block Attention Module (CBAM) is independently applied to both the RGB image and its corresponding segmentation mask to emphasize important visual and spatial features. Enhanced features are fused and fed into a lightweight MobileNetV2 classifier for disease severity prediction. RCAMNet achieved a test accuracy of 96.23%, outperforming conventional raw image-based models (89.58%). Interpretability is enhanced through Grad-CAM visualizations. RCAMNet demonstrates robust performance in classifying BLB severity across diverse environmental conditions, confirming its real-world deployment potential. Additionally, the proposed framework supports the development of edge device-compatible solutions, enabling real time monitoring and improved disease management in precision agriculture.

## Introduction

Rice cultivation is the backbone of agro-based economies around the world. It is essential for the food and economic well-being of people in regions such as southern India, which are engaged in significant rice production. However, several diseases pose great challenges to rice production; one of them is bacterial leaf blight (BLB), a harmful rice disease caused by the bacterium Xanthomonas oryzae pv. oryzae Xoo^1^. The disease causes 20 to 50 percent crop losses in extremely affected regions and is spread by contaminated agricultural equipment, flooding, and infected or vulnerable seedlings^2^. BLB is most common in tropical and subtropical areas with favorable climatic conditions for the development of the disease. Options such as planting BLB resistant varieties, adopting cultural practices, and using biological control agents are important for field-specific BLB management. Changes in ecosystems and the deterioration of soil quality further aggravate the disease, resulting in less yield. The rate of increase in the spread of bacterial leaf blight in rice fields in Kerala has been reported to be high^3^. This increase might be due to the spread of the pathogen by flood waters. In addition, flood-related changes in soil characteristics and climate could also have caused a rise in both the incidence levels and severity of the disease^4^. Although resistant cultivars are necessary for disease mitigation, early and proper detection and diagnosis are critical to effective disease management. However, traditional BLB detection methods that rely on the visual inspection of the farmer are often subjective, time consuming, and prone to errors, especially in the early stages of infection^5^. Laboratory analysis is the traditional method for diagnosing the progression of BLB, which is time-consuming and requires well-equipped laboratories, making it less practical for early stage disease detection. Pathogen strains that cause diseases are often isolated in controlled laboratory settings, and their DNA or RNA information is retrieved and compared with the Gene Bank database to determine the stage of the disease^6^. To address the aforementioned challenges, the primary objective of this study is to develop a comprehensive data set focused on analyzing the progression of rice BLB disease and to propose an improved multitasking deep learning approach that combines segmentation and classification strategies. This approach aims to precisely identify and assess the severity of BLB disease in rice leaves while effectively capturing the different stages and backgrounds of the disease. The main contributions of the proposed work include the following:

- Development of the BLBVisionDB dataset to analyze the progression of rice BLB disease, consisting of high-quality images collected from infected paddy fields in Kerala and artificially inoculated plants grown under controlled greenhouse conditions.
- We systematically curated the BLBVisionDB dataset, classifying it into five distinct categories based on the Leaf Area Infected (LAI) and included precise manual annotations under expert supervision to ensure its accuracy, reliability, and suitability for both segmentation and classification tasks, as well as advanced disease progression analysis.
- We propose RCAMNet, a multitask deep learning approach that integrates segmentation and classification for the analysis of BLB diseases. Using Detectron2 for multiclass segmentation, the model identifies regions of interest (ROI) to monitor disease stages. The Convolutional Block Attention Module (CBAM) enhances feature representation, improving classification performance. The refined features are processed through the MobileNetV2 backbone to classify the severity of the BLB disease into five stages, offering a comprehensive solution for the analysis of disease progression.
- Conduct a comprehensive analysis of supervised segmentation models, including DeepLabv3, Multiclass U-Net, and Detectron2, evaluating their ability to generate segmentation masks and identify regions of interest (ROI) for BLB pathogenesis. Additionally, a detailed analysis of the classification performance of the MobileNetV2 model using three approaches: raw image classification without attention, where MobileNetV2 is applied directly to the input images; image classification with the CBAM attention module, where the CBAM module enhances the extracted features; and image classification with Detectron masks and CBAM features.

### Literature review

The existing literature primarily spans two main themes: deep learning solutions for plant disease analysis, and the incorporation of attention mechanisms to enhance model performance.

#### Deep learning solutions for plant disease analysis

Deep learning techniques, including segmentation-based methods and convolutional neural networks, are emerging as powerful tools to detect plant diseases and assess their severity. The study^7,8^ examines several deep learning approaches, such as convolutional neural networks (CNNs) and their use to detect and classify plant diseases from photographs, demonstrating the wide application potential of CNN-based models in agriculture. Several studies have explored the application of deep learning in plant disease detection and classification. A comprehensive evaluation of the most recent state-of-the-art deep learning approaches for plant disease detection and classification has been presented, highlighting their potential to revolutionize agricultural practices^9^. An in-depth overview of the diagnostics of rice plant disease using machine learning approaches has been provided, with a focus on recent advances and methodologies in the field^10^. The AgriDet framework, developed to address challenges such as occlusion and low-quality backdrops, combines INC-VGGN and Kohonen-based networks^11^. A multilevel handcrafted feature extraction technique has been introduced, fusing color texture (LBP) and color features (CC) to achieve high accuracy in identifying multiple rice diseases^12^.

A method has been proposed to classify rice leaf diseases based on its chroma and segmented boundaries, employing a coarse to fine classification strategy to improve accuracy^13^. Contributions to this domain include the development of a deep learning system for early disease identification using advanced image analysis techniques^14^. The MobileNetV2-Inception model has been shown to provide a lightweight yet effective solution for the classification of rice plant diseases by integrating MobileNetV2 with an Inception module to improve both efficiency and performance^15^. High segmentation accuracy for agricultural applications has been achieved using an attention residual U-Net^16^. The MC-UNet framework, introduced for tomato leaf disease segmentation, demonstrates the power of multi scale convolution in achieving superior results^17^. Detectron2 has demonstrated remarkable versatility in medical applications, such as the detection of lesions in diabetic retinopathy, where a fine-tuned MR-CNN (R50-FPN) model achieved a segmentation accuracy of 99.34% for hemorrhages^18^. A novel multi-label identification framework (LDI-NET) using CNN and transformers has been proposed to simultaneously identify plant type, leaf disease, and severity, effectively leveraging local and global contextual features for accurate disease identification^19^. A hybrid inception-xception convolutional neural network (IX-CNN) has been introduced for efficient plant disease classification and detection, achieving high accuracy across various datasets^20^.

#### Attention mechanism for enhancing deep learning models

Attention mechanisms play a crucial role in deep learning models by allowing them to concentrate on the most relevant features in input data, which improves both accuracy and readability. These techniques dynamically give weights to crucial sections or characteristics of the data, making them particularly useful in applications like image segmentation, natural language processing, and disease detection. Attention mechanisms have been widely applied across various domains to improve the performance of deep learning models. For instance, an attention-guided CNN framework has been proposed to classify breast cancer histopathology images, using a supervised attention method to focus on regions of interest^21^. Similarly, a combined attention mechanism with a multi-scale latent representation system has been introduced to isolate essential areas of interest and generate precise attention maps for improved analysis^22^. In the field of plant disease identification, a modified CBAM attention module has been designed to enhance CNN recognition rates, resulting in significant performance improvements^23^. A recent study presents a tomato leaf disease detection approach employing the Convolutional Block Attention Module (CBAM) and multiscale feature fusion, demonstrating considerable increases in detection performance^17^. Advanced attention mechanisms and residual connections have been utilized in the development of the Interpretable Attention Residual Skip Connection SegNet (IARS SegNet) for melanoma segmentation, aiming to improve segmentation accuracy while maintaining model interpretability^24^. Fusion models employ attention processes to improve precision and robustness in classification tasks by combining data from different sources. For example, CofaNet, a classification network that combines CNNs and transformer-based fused attention, has been introduced to achieve state-of-the-art performance^25^. A hybrid model for multi-modal image fusion, employing a convolutional encoder and a transformer decoder, has demonstrated significant qualitative improvements^26^. Recently, the MD-Unet, a multi-scale residual dilated segmentation model, has been developed to enhance the segmentation of tobacco leaf lesions. By integrating attention mechanisms and improving feature extraction, the MD-Unet achieved high accuracy and efficiency, outperforming several existing models in segmenting various tobacco diseases^27^.

Although there have been substantial advances in plant disease detection and classification, there is still a noticeable gap in the early-stage disease detection and severity classification of BLB disease in rice. BLB usually begins in localized leaf areas, so early detection is crucial for timely intervention and prevention. However, training models on such regional variances is challenging due to a lack of labeled datasets monitoring BLB advancement. Most present models concentrate on disease diagnosis at advanced stages. The primary aim of this research is to develop a model capable of detecting bacterial blight disease in rice in its early stages and categorizing its severity, facilitating timely prevention using a combination of segmentation and deep learning techniques. This approach seeks to address the current limitations and improve the accuracy and efficiency of BLB pathogenesis monitoring.

The paper is structured as follows: Section 1 elaborates on the dataset collection process, Section 2 outlines the experimental design of RCAMNet, and Section 3 discusses the experimental results. Finally, Section 4 presents a discussion of the results, including their implications, limitations, and potential future research directions, followed by Section 5 which concludes the study.

## BLBVisionDB: Rice BLB Severity Dataset for Classification and Segmentation

In this section, we present the development of BLBVisionDB, a comprehensive dataset for the classification and segmentation of rice bacterial leaf blight (BLB) severity.

### Data acquisition through field sampling and artificial inoculation

The BLBVisionDB dataset comprises images captured from real paddy fields across Kerala under diverse conditions, including varying lighting (bright sunlight, cloudy skies, and shadows), different stages of disease severity (from early symptoms to advanced lesions), and natural occlusions (such as overlapping leaves or weeds). In addition, the dataset includes images generated through artificial inoculation to ensure controlled and standardized representations of disease progression with uniform lighting and clean backgrounds. To induce BLB under controlled conditions, a virulent bacterial strain was selected and rice plants were cultivated in a regulated environment. The bacterial suspension was uniformly sprayed onto the leaves, followed by incubation under optimal conditions to promote infection. Symptoms were carefully monitored and documented at different stages of progression to ensure consistent and reproducible data.

**Fig. 1:**
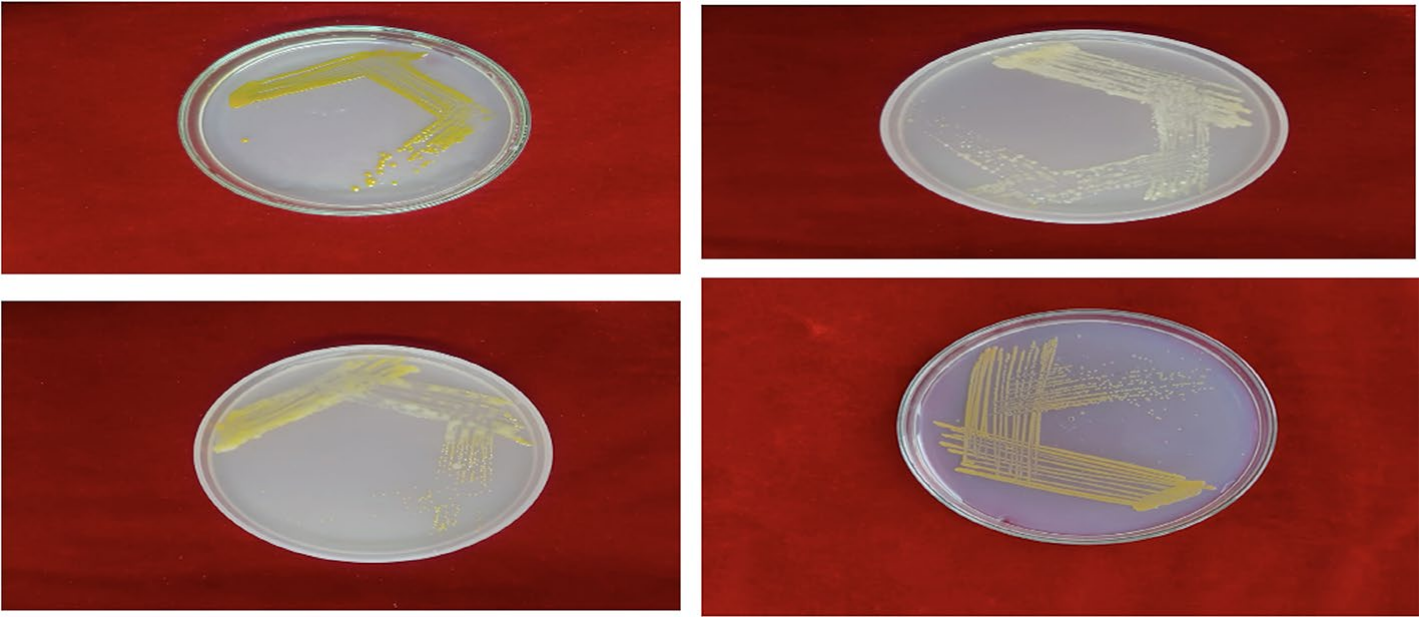
Colonies of *Xanthomonas* isolated from diseased leaf samples.

**Fig. 2:**
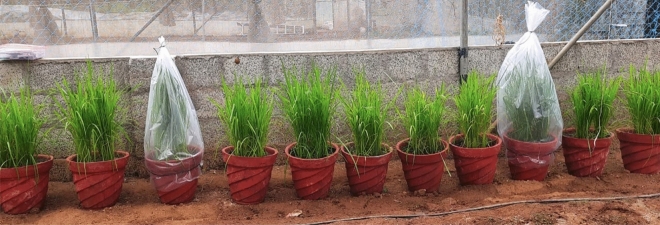
Controlled pot culture experiment in a polyhouse for artificial inoculation.

1. **Collection and isolation of pathogen:** Diseased leaf samples were collected from paddy fields in Palakkad, Thrissur, and Malappuram districts of Kerala, with the support of trained personnel from the Department of Plant Pathology, Kerala Agricultural University. The samples were brought to the laboratory, where the presence of BLB was confirmed and the causative bacterium, *Xanthomonas oryzae*, was isolated and purified through culturing on nutrient agar as illustrated in Fig. 1.
2. **Experimental setup, inoculation, and image capture:** Rice seeds of the variety *Uma*, highly susceptible to BLB, were cleaned, disinfected, and sown into ten pots arranged in a polyhouse with a north–south orientation for optimal sunlight. After germination, the plants were artificially inoculated with the isolated pathogen to induce disease symptoms. Plastic sheets were used to maintain a controlled, humid environment conducive to infection, as shown in Fig. 2. As symptoms progressed, a standardized protocol was followed to capture high-resolution images: six tagged leaves per plant were photographed in optimal daylight using a 64-megapixel mobile camera, ensuring consistent timing, distance, and framing as per Table 1. Approximately 100 high-quality images were collected and subsequently graded by experts for severity assessment. The images were then curated and labeled for severity on a 0–7 scale, based on expert assessment. These severity labels form a robust foundation for training and evaluating models on BLB disease progression. The grading scheme is summarized in Table 2.

**Table 1:** Experimental setup for artificial inoculation process.

Parameter	Details
Rice Variety	Uma (BLB-susceptible)
Polyhouse Size	300 m^2^
Lighting Orientation	North-South
Inoculation Method	Wound and spray
Monitoring Frequency	Daily
Camera Resolution	64 megapixels

**Table 2:** Scoring system used to evaluate the BLB disease progression.

Stage	Lesion Length (cm)	Features Considered	Diseased Area (%)	Description
Healthy	0	- Uniform green color, smooth leaf texture - No lesions; veins appear clear	0	Healthy
Stage 1 (Incubation)	<3	- Tiny, water-soaked, translucent lesions - Slight chlorosis possible	1–5	Resistant
Stage 2 (Expansion)	3–6	- Lesions expand; more numerous - Light brown or grayish color	5–10	Moderately Resistant
Stage 3 (Sporulation)	6–12	- Lesions merge; form brown patches - Affects much of leaf area	10–20	Moderately Susceptible
Stage 4 (Sporulation)	12–24	- Large merged lesions - Necrosis, wilting begins	24–40	Susceptible
Stage 5 (Wilting)	>24	- Necrosis covers most of leaf - Wilting prominent	>40	Susceptible

*Source: Standard Evaluation System of Rice (IRRI, 1988)*.

### Manual dataset annotation for multiclass segmentation

To ensure the dataset’s suitability for advanced tasks like multiclass segmentation, we annotated each image by marking diseased areas and categorizing them by severity of infection. This is crucial for pixel-level segmentation of rice leaves, enabling differentiation among the five BLB stages and exclusion of irrelevant background elements. This dataset, detailed in Table 3, comprehensively covers the entire progression of BLB with detailed annotations. The final dataset, exported in COCO format, ensures compatibility with popular segmentation models.

**Fig. 3:**
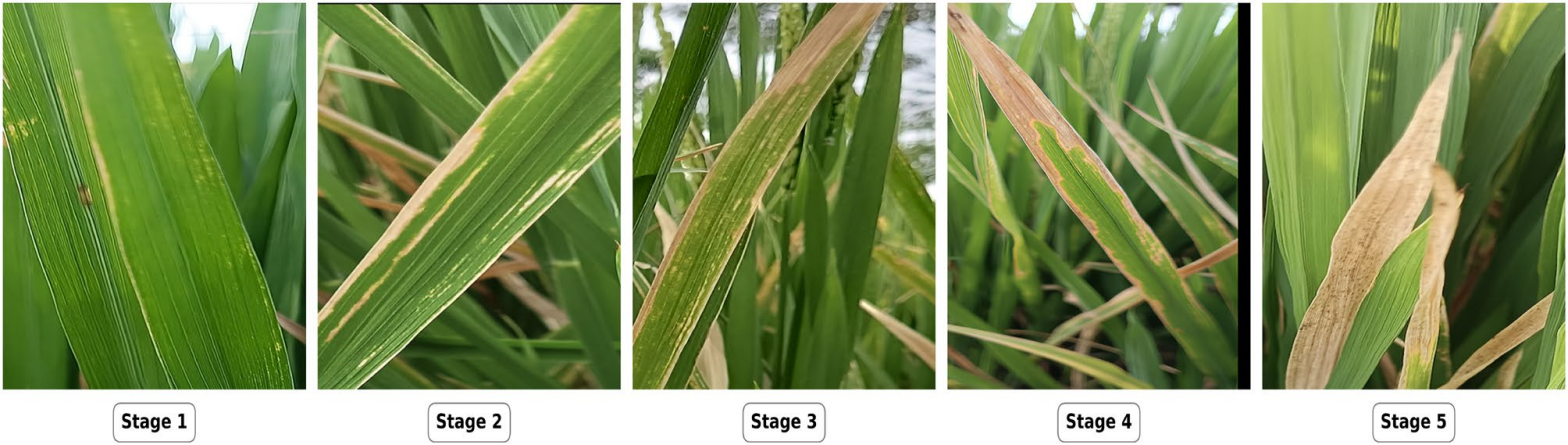
Sample images representing various stages of rice bacterial leaf blight disease.

#### Data augmentation techniques

Various data augmentation approaches were used during preprocessing to increase the adaptability of the proposed model and to simulate various environmental conditions encountered in real paddy fields. These conditions include inconsistent lighting, complex backgrounds, and image noise. The techniques used are horizontal flipping (p = 0.5), brightness adjustment (±30%), contrast modification (±20%), random rotation ($\pm {15}^\circ$), and Gaussian noise addition. These enhancements were applied uniformly in all five stages of disease severity. By artificially expanding the training dataset with such diverse variations, we aim to improve the ability of the model to detect disease symptoms more accurately and reliably in challenging real-world scenarios. Sample images from the *BLBVisionDB* for classification are shown in Fig. 3. Original images were resized to 300 $\times$ 300 pixels, and augmentation increased the size of the data set to 5,896 images. These were divided into training (4540), validation (905), and testing (451) sets, as detailed in Table 3. Our *BLBVisionDB* dataset used in this work has been uploaded to https://sudheshkm.github.io/Bacterial-Leaf-Blight-disease-progression/ and is available on reasonable request.

**Table 3:** Number of images in each stage from the dataset.

BLB_Stage	Training	Validation	Testing
Stage_1	914	182	91
Stage_2	1137	227	113
Stage_3	957	191	95
Stage_4	779	155	77
Stage_5	753	150	75
**Total**	**4540**	**905**	**451**

## RCAMNet: Multi-Task Model Design for BLB Disease Severity Analysis

**Fig. 4:**
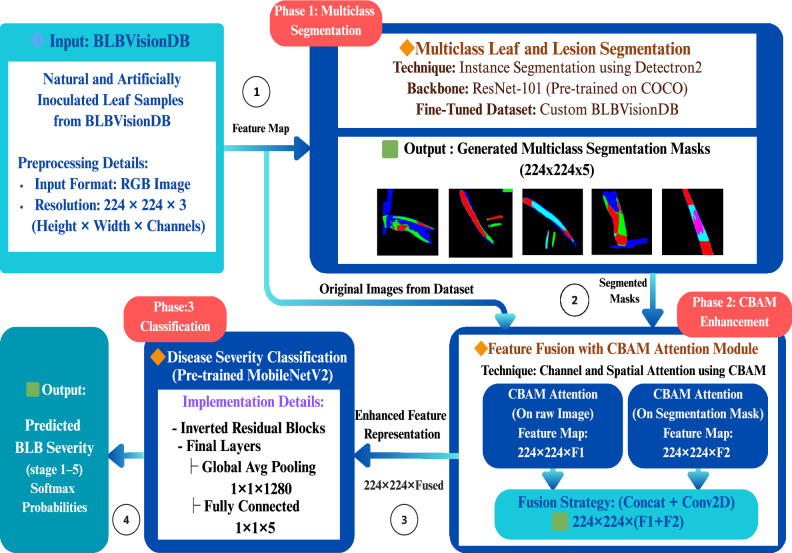
The proposed RCAMNet pipeline for rice BLB severity analysis which integrates raw RGB image features and multiclass segmentation masks to emphasize disease affected regions. These two feature streams are enhanced using CBAM Modules and fused before being passed to a lightweight MobileNetV2 classifier for final severity stage prediction.

This section introduces the proposed multitask model, **RCAMNet** (Rice Classification with Attention Model Network), designed in three phases to assess the progression of rice bacterial leaf blight disease (BLB). The detailed architectural flow diagram of the proposed model is illustrated in Fig. 4. The first phase generates **multiclass segmentation mask** to identify and localize disease affected regions at the pixel level. In the second phase, the **Convolutional Block Attention Module (CBAM)** is utilized to enhance feature extraction by refining both the **raw image features** (the original RGB input image) and the **multiclass segmentation mask** (multiclass pixel wise labels). The final phase employs **MobileNetV2** for classification, using the fused representation of the raw image features and multiclass segmentation masks to improve accuracy and provide a robust solution for BLB severity analysis. Throughout this section, we use the following terms consistently:

- **Raw image features:** The original RGB pixel values of the input rice leaf images, represented as tensors of shape $H \times W \times 3$.
- **Multiclass segmentation mask:** Multiclass, pixel-wise labels generated by the segmentation model, identifying and localizing diseased regions in the image; these have the same spatial dimensions as the input image.
- **Enhanced features:** The refined and fused representation of raw image features and multiclass segmentation masks after CBAM attention processing.

### Phase 1: Multiclass segmentation using detectron2

We employed **Detectron2**, a modular deep learning framework for object detection and segmentation, to perform multiclass BLB disease segmentation. Its object-centric methodology systematically recognizes individual components such as rice leaves and classifies them based on distinguishing features.

Let $D = \{(x_i, y_i, m_i)\}_{i=1}^N$ denote the custom BLBVisionDB dataset, where each sample consists of an input image $x_i$ (raw image features), its corresponding severity label $y_i$, and the multiclass segmentation mask $m_i$. The input images $x_i$ are tensors of shape $\mathbb {R}^{H \times W \times C}$, where *H* and *W* denote the height and width of the image, and $C=3$ represents the RGB channels. The severity labels $y_i$ are scalar values in $\mathbb {Z}$ within the range [0, *K*], where $K=5$ corresponds to the five stages of BLB severity. Multiclass segmentation masks $m_i$ are pixel-wise labels of the same spatial dimensions as $x_i$, highlighting the affected regions. Thus, each dataset sample provides both a severity label and a multiclass segmentation mask, serving as the foundation for the segmentation and classification tasks. The first task of the proposed multi-task model, the segmentation task, is formulated as:

1 $$\begin{aligned} \hat{m}i&= f{\text {seg}}(x_i) \end{aligned}$$

where $f_{\text {seg}}$ denotes the segmentation model, and $\hat{m}_i$ is the predicted multiclass segmentation mask for the input image $x_i$. The model is trained in a supervised fashion, using the corresponding multiclass ground truth segmentation mask $m_i$. To produce these masks, Detectron2 leverages a Region Proposal Network (RPN) to identify candidate object regions. These regions are processed through ROI pooling and subsequently passed to the mask prediction head, which generates multiclass pixel masks. This process can be formally described as:

2 $$\begin{aligned} M = \text {MaskHead}(\text {ROI}\_\text {pooling}(\text {RPN}(x_i; \theta _{\text {RPN}}); \theta _{\text {ROI}}); \theta _{\text {Mask}}) \end{aligned}$$

where $\theta$ (with relevant subscripts) represents the learnable parameters of each module within the Detectron2 pipeline: the Region Proposal Network ($\theta _{\text {RPN}}$), the ROI pooling layer ($\theta _{\text {ROI}}$), and the Mask Head ($\theta _{\text {Mask}}$). We initialized the segmentation model with a pre-trained mask_rcnn_R_101_FPN_3x checkpoint from the Detectron2 Model Zoo, enabling effective transfer learning for BLB severity segmentation. Furthermore, the model was adapted for multiclass semantic segmentation, configured to predict five classes corresponding to the five severity stages of BLB. This design effectively integrates features at the image and region levels, improving the model’s ability to discern subtle differences between severity stages. Table 4 provides the detailed hyperparameter settings. The segmentation output serves as rich spatial and contextual input to the classification task, enabling precise severity analysis.

**Table 4:** Hyperparameters for Detectron2.

HyperParameter	Value
Configuration File	mask_rcnn_R_101_FPN_3x
Data Loading Workers	4
Mask Prediction	True
Base Learning Rate	0.00025
Max Iterations	3000
Proposals per Image	128
Learning Rate Decay	Disabled
Number of Classes	5

### Phase 2: Feature enhancement with CBAM attention module

**Fig. 5:**
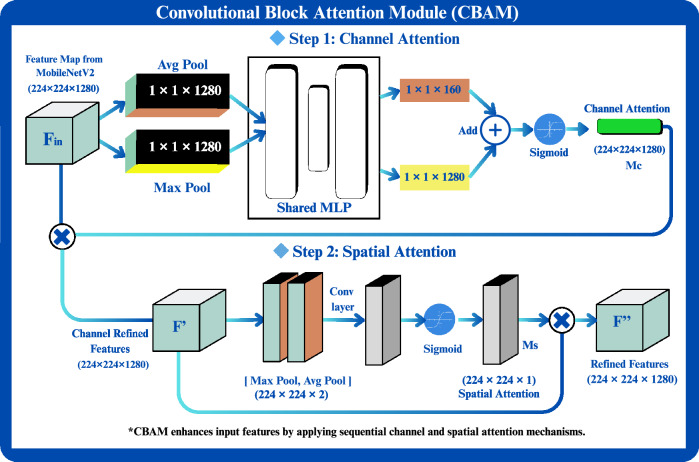
Internal architecture of the Convolutional Block Attention Module (CBAM) used in the proposed pipeline.

To enhance the discriminative capacity of the model, RCAMNet integrates the Convolutional Block Attention Module (CBAM)^28^ into the feature extraction pipeline. Unlike conventional approaches in which CBAM is applied to a single input stream, the proposed model applies CBAM independently to both the original RGB image and the corresponding multiclass segmentation mask (generated in Phase 1). This dual-pathway attention mechanism enables the network to exploit complementary information: visual textures from the RGB image and lesion-specific spatial patterns from the multiclass segmentation mask. Figure 5 shows a block-level representation of the integrated CBAM module within the proposed pipeline, illustrating both channel and spatial attention mechanisms applied to the input features. The attention-augmented outputs from the RGB image and segmentation mask streams are subsequently concatenated and passed through a convolutional layer to fuse the features into a unified representation. This fused feature map is then forwarded to the final classification module (Phase 3) to predict the severity stage of BLB. This attention-guided fusion allows the model to selectively focus on the most relevant regions and modalities, thereby improving classification performance, particularly for disease stages exhibiting subtle lesion characteristics.

### Phase 3: Classification with MobileNetV2

**Phase 3** focuses on classifying the severity stages of BLB using CBAM-enhanced features extracted from both the raw images ($\textbf{F}_{\text {image}}$) and the corresponding segmentation masks ($\textbf{F}_{\text {mask}}$). These features are concatenated along the channel dimension to form a unified representation: to produce the combined feature map **along the channel dimension** ($C$)

3 $$\begin{aligned} \hat{\textbf{y}} = \text {FC} \left( \text {GAP} \left( \text {Conv} \left( \text {Concat}(\textbf{F}_{\text {image}}, \textbf{F}_{\text {mask}}, \text {dim}=C) \right) \right) \right) \end{aligned}$$

Here, $\text {Conv}(\cdot )$ denotes a learnable convolutional layer that refines the merged features, $\text {GAP}(\cdot )$ is Global Average Pooling to reduce spatial dimensions, and $\text {FC}(\cdot )$ denotes the final classification layer. The fused features are fed into a MobileNetV2 backbone, where the final layer outputs class logits $\hat{\textbf{y}} \in \mathbb {R}^K$, with $K = 5$ representing the five stages of severity of BLB.

The classification model is optimized using the cross-entropy loss function:

4 $$\begin{aligned} \mathcal {L}_{\text {classification}} = -\frac{1}{N} \sum _{i=1}^{N} \sum _{k=1}^{K} y_{i,k} \log (\hat{y}_{i,k}) \end{aligned}$$

where $N$ is the total number of samples, $y_{i,k}$ is the ground truth label for class $k$ for sample $i$ (encoded as a one-hot vector), $\hat{y}_{i,k}$ is the predicted probability for class $k$ for sample $i$. By integrating CBAM-enhanced features from both images and masks. The use of cross-entropy loss promotes effective optimisation and improves the model’s generalisability.

## Experimental Results and Discussion

This section presents a comprehensive analysis of the experimental results to assess the effectiveness and performance of the proposed multitask model. The results are systematically organized into three key aspects:

1. Evaluation of supervised segmentation backbones.
2. Impact of Attention Mechanisms on Feature Enhancement.
3. Classification Performance Using MobileNetV2.

### Evaluation of supervised segmentation models

This subsection focuses on evaluating supervised segmentation models for generating multiclass segmentation masks of BLB. We compared the effectiveness of three state-of-the-art segmentation models from the literature: Multiclass U-Net, DeepLabv3, and Detectron2. The performances of these models were assessed using the Mean Intersection-over-Union (MIOU) metric, which is commonly used in segmentation tasks to compute the average overlap between the predicted segmentation masks and the ground-truth masks across all classes.

In this analysis, we focus specifically on the disease symptom regions, as these areas are critical for identifying the progression of BLB. Table 5 tabulates the performance comparison of three segmentation pillars considered in the study. The Detectron2 model achieved the highest MIOU of 59.49%, outperforming other methods. This superior performance can be attributed to Detectron2’s ability to capture multiscale features, which is essential for accurately delineating disease symptom pixels within a single rice leaf image. The segmentation results provide a solid foundation for subsequent feature enhancement and classification tasks. However, even with superior segmentation results, difficulties persist due to the complexity of disease recognition. These complications result from the similarity and patterns of diseases present in various stages of rice blast disease (BLB), as well as the existence of cluttered backgrounds.

**Table 5:** Average IoU for segmentation models. The model with the highest MIOU is highlighted in bold.

Model	Average IoU (%)
Multiclass Unet	32.63
Deeplabv3	47.05
**Detectron2**	**59.49**

**Table 6:** Color to label conversion.

Color (BGR)	Class Label
(255, 0, 0) - (Blue)	BLB Stage 1
(0, 255, 0) (Green)	BLB Stage 2
(0, 0, 255) - (Red)	BLB Stage 3
(255, 255, 0) - (Yellow)	BLB Stage 4
(255, 0, 225) - (Magenta/Fuchsia)	BLB Stage 5

**Fig. 6:**
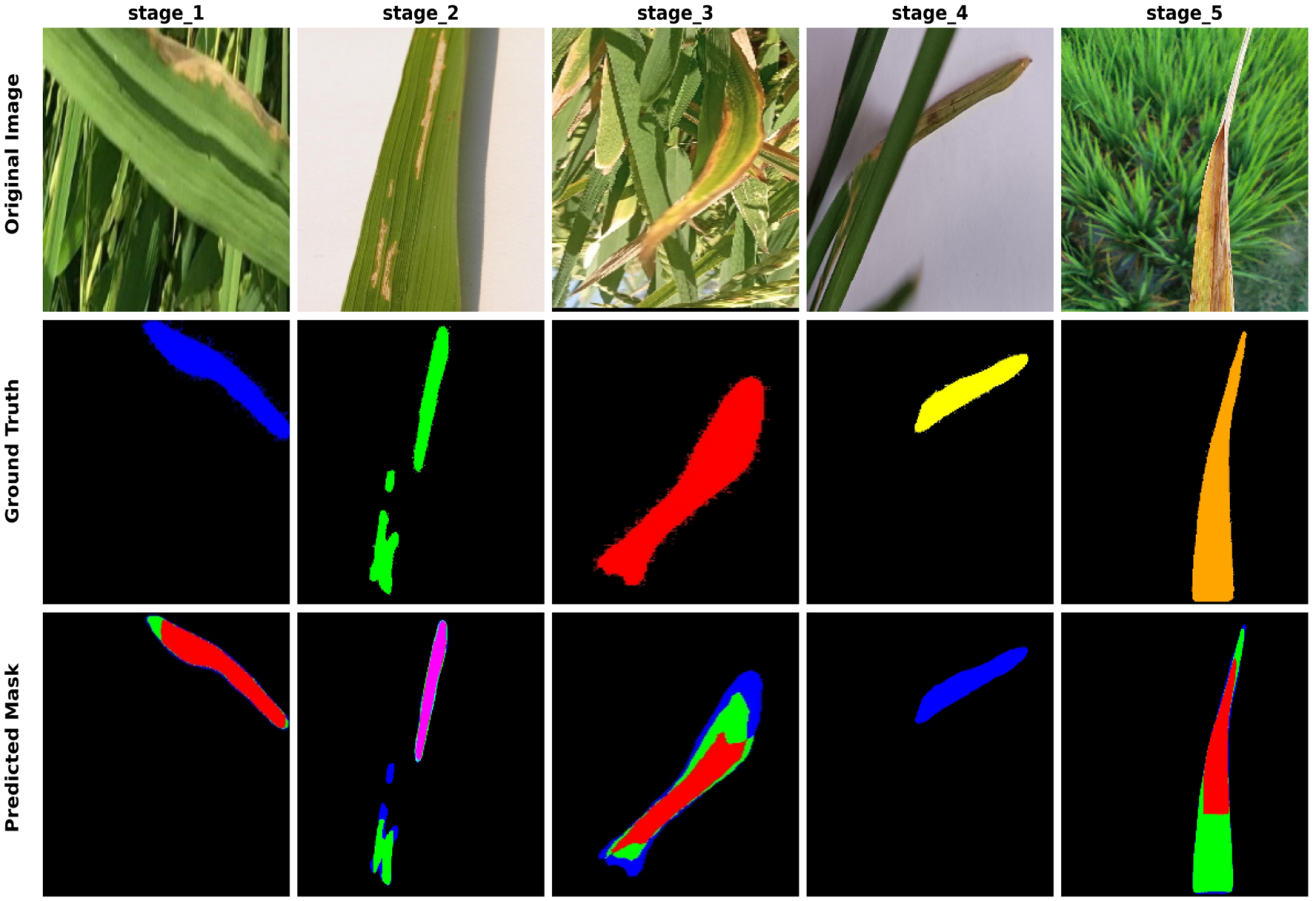
Rice bacterial leaf blight images and Detectron2 mask.

Figure 6 represents sample images of the segmentation output obtained using detectron2. The first row showcases the original images representing various stages, while the second row illustrates their corresponding segmentation results. The color scheme employed in the segmentation aligns with the color-to-label conversion outlined in Table 6. Our observations demonstrate Detectron2’s effectiveness in detecting different stages of rice BLB disease. In particular, as the disease progresses from Stage_1 to Stage_5, small color changes become apparent, ranging from bright yellow to slightly darker colors, eventually laying out as brown in Stage_5. The segmentation results demonstrate the model’s ability to appropriately determine stage development.

### Classification performance of CNN Models using CBAM attention

In our previous study on rice leaf disease identification using transfer-learned DCNNs, we demonstrated that deep learning, particularly transfer learning with DCNNs, is highly effective for plant disease detection^29^. Based on these findings, we extended our research to assess the progression of rice BLB disease using similar methodologies. For this study, we chose MobileNetV2 as the primary model due to its lightweight architecture and superior performance in terms of accuracy and computational efficiency. This decision reflects the need for an optimal balance between precision and resource utilization in real-world agricultural applications. The analysis is carried out in three steps. First, we classify five distinct severity stages of BLB (stage_1 to stage_5) using only raw images from the *BLBVisionDB* dataset, bypassing a segmentation module. Secondly, the effect of incorporating the CBAM attention mechanism into the raw image features on disease severity detection is evaluated. Finally, we examine the combined effect of integrating both the multiclass segmentation module and the CBAM. This step-by-step approach highlights the benefits of incrementally building our proposed model for improved BLB stage classification.

**Fig. 7:**
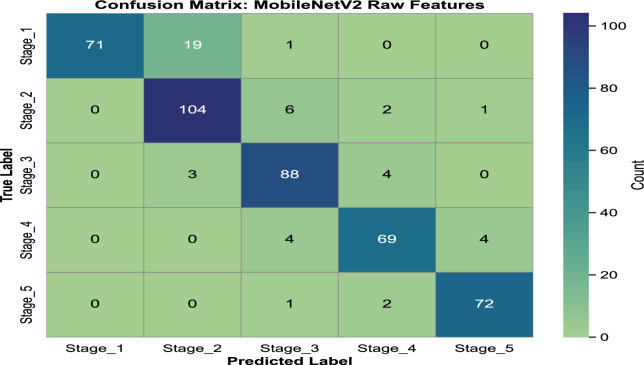
Confusion matrix for raw image classification using MobileNetV2.

**Fig. 8:**
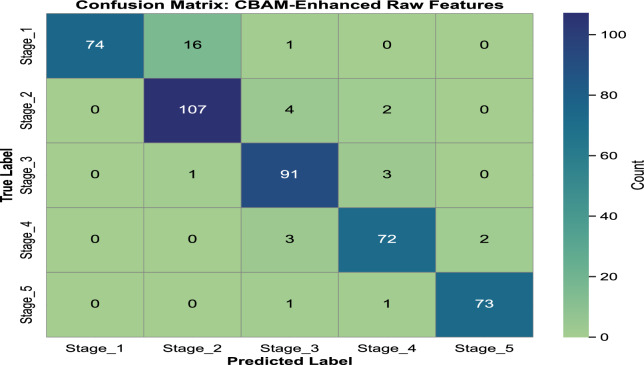
Confusion matrix for MobileNetV2 with CBAM-enhanced row image features.

**Fig. 9:**
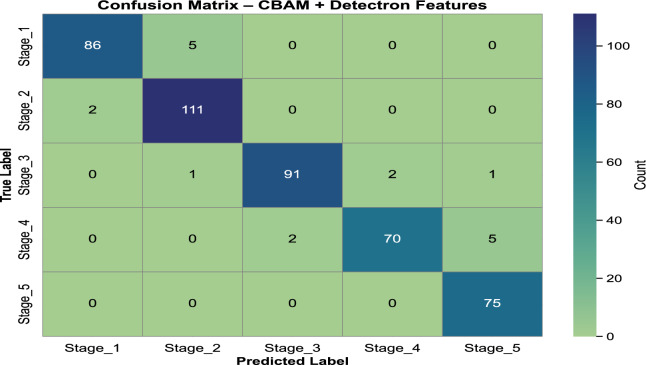
Image classification with CBAM-enhanced row image features and detectron mask features.

**Fig. 10:**
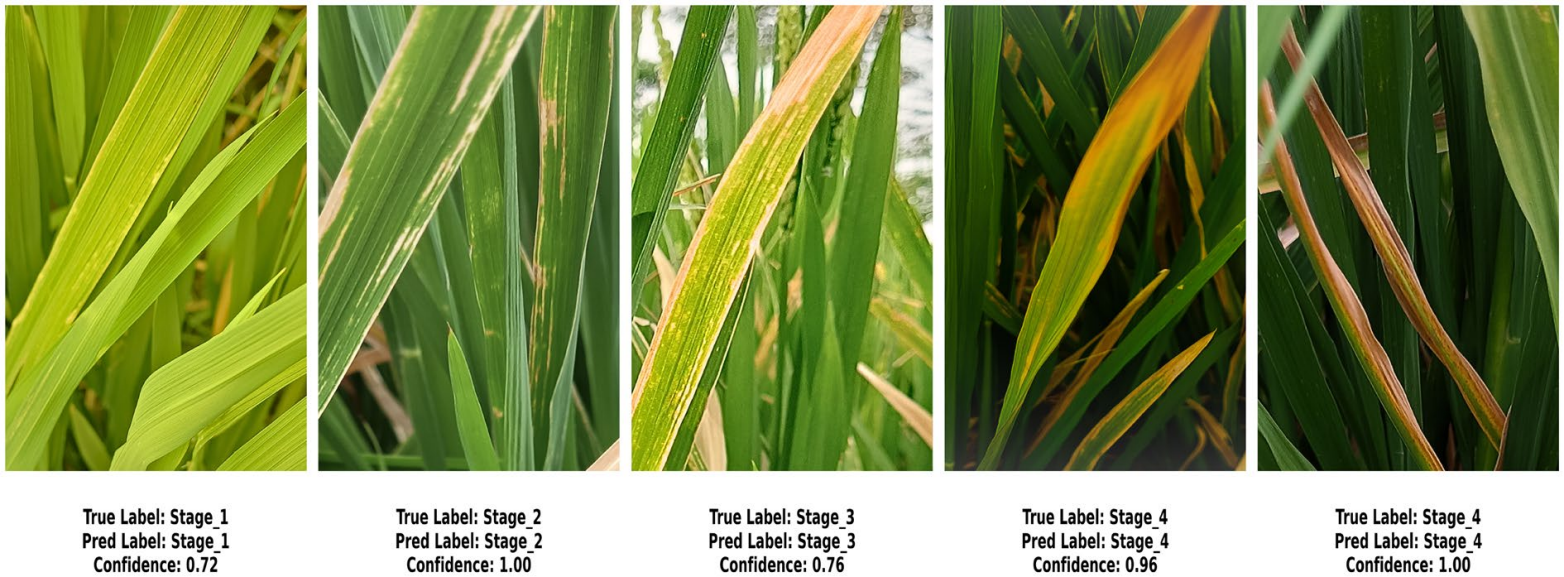
Qualitative prediction results of RCAMNet on real-world test images captured under challenging conditions such as occlusion, complex foliage, and lighting variations.

1. **BLB stage classification from row images without attention and segmentation:** In the first set of experiment, we evaluated the performance of the MobileNetV2 model without including any attention mechanisms. The pre-trained weights of MobileNetV2 were used as a baseline, with the final classification layer replaced by a fully connected layer containing five neurons corresponding to the severity stages of BLB. Categorical cross-entropy was used as a loss function, and a dropout ratio of 0.5 was applied to prevent overfitting. MobileNetV2 achieved a test accuracy of **89.58%**, precision of **90.24%**, recall of **89.58%**, and F1-score of **89.56%**. The confusion matrix for this approach is illustrated in Fig. 7. Notable misclassifications include 21.1% of stage_1 instances being misclassified as stage_2 and 5.4% of stage_2 instances predicted as stage_3. Similarly, 4.3% of stage_3 cases are misclassified as stage_4, while 5.5% of stage_4 instances are misclassified as stage_5. These misclassifications indicate that the model has difficulty distinguishing between adjacent stages, likely due to overlapping features or subtle differences in their characteristics.
2. **Ablation study: impact of data augmentation** To examine the contribution of data augmentation to classification accuracy, we conducted an ablation study comparing model performance on raw images with and without augmentation. The same backbone architecture (MobileNetV2 without CBAM or segmentation) was used across both configurations to ensure fair comparison. The class-wise results, including precision, recall, and F1-score for each severity stage, are presented in Table 7 and Table 8. These results demonstrate a substantial improvement in classification performance, attributable to the increased variation and robustness introduced through augmentation.
3. **BLB classification with CBAM features:**In this approach, the CBAM attention mechanism was integrated to enhance raw image features. By applying both channel and spatial attention to the input images, the CBAM module improved feature extraction, leading to enhanced performance. The model achieved a test accuracy of **92.46%**, precision of **93.39%**, recall of **92.53%**, and F1-score of **92.71%**. The corresponding confusion matrix is shown in Fig. 8. The integration of the CBAM attention mechanism has significantly reduced misclassification rates across all stages. For instance, misclassification from Stage_1 to Stage_2 decreased from 21.1% to 17.8%, and from Stage_4 to Stage_5, it dropped from 5.2% to 2.6%. Similarly, Stage_2 to Stage_3 errors were reduced from 5.3% to 3.5%, and Stage_3 to Stage_4 from 4.2% to 3.2%. These reductions highlight CBAM’s ability to enhance feature extraction, leading to improved classification performance, as reflected in the higher overall accuracy and F1-score.
4. **Image classification with CBAM-enhanced row image features and detectron mask features:** In the third strategy, we further improved the model’s performance by combining the raw image features with the segmentation masks generated by the Detectron2 model. The masks were passed through the CBAM module along with the raw input to highlight diseased regions more effectively. This combined approach significantly improved the classification results, achieving a test accuracy of **96.23%**, precision of **95%**, recall of **95%**, and F1-score of **95%**. The confusion matrix for this enhanced method is illustrated in Fig. 9. The integration of the Detectron2 segmentation module and CBAM significantly reduced misclassification rates compared to the baseline model. For Stage 1, misclassification decreased from 21.1% to 8.79%, while for Stage 2, it dropped from 5.4% to 1.77%. Similarly, Stage 3 errors were reduced from 4.3% to 2.15%. Although Stage 4 showed a slight increase in misclassification from 5.5% to 9.46%, Stage 5 maintained a perfect classification rate of 0%. These improvements demonstrate the effectiveness of the enhanced architecture in capturing finer details and improving classification accuracy across most stages.
5. **RCAMNet Performance in Complex Environments:** To evaluate the generalization capability of RCAMNet under practical deployment scenarios, we tested the model on challenging images exhibiting conditions such as low lighting, partial occlusions, and cluttered natural backgrounds. Figure 10 presents qualitative results, where the model consistently predicts the correct BLB severity stages with high confidence across diverse environmental variations. These results highlight RCAMNet’s robustness and adaptability in real-world field conditions, even when visual symptoms are partially obscured or poorly illuminated.

**Table 7:** Classification performance for each BLB severity stage using MobileNetV2 on raw images.

Stage	Precision	Recall	F1-Score
Stage 1	80	75.5	77.7
Stage 2	77.3	79.2	78.4
Stage 3	78.5	77.2	77.7
Stage 4	75.2	74.6	74.6
Stage 5	79.1	78.5	78.7

**Table 8:** Classification performance for each BLB severity stage using MobileNetV2 on augmented images.

Stage	Precision	Recall	F1-Score
Stage 1	92.3	88.1	90.1
Stage 2	89.5	91.2	90.3
Stage 3	90	89.5	89.7
Stage 4	88.7	87.9	88.3
Stage 5	90.8	91	90.9

**Fig. 11:**
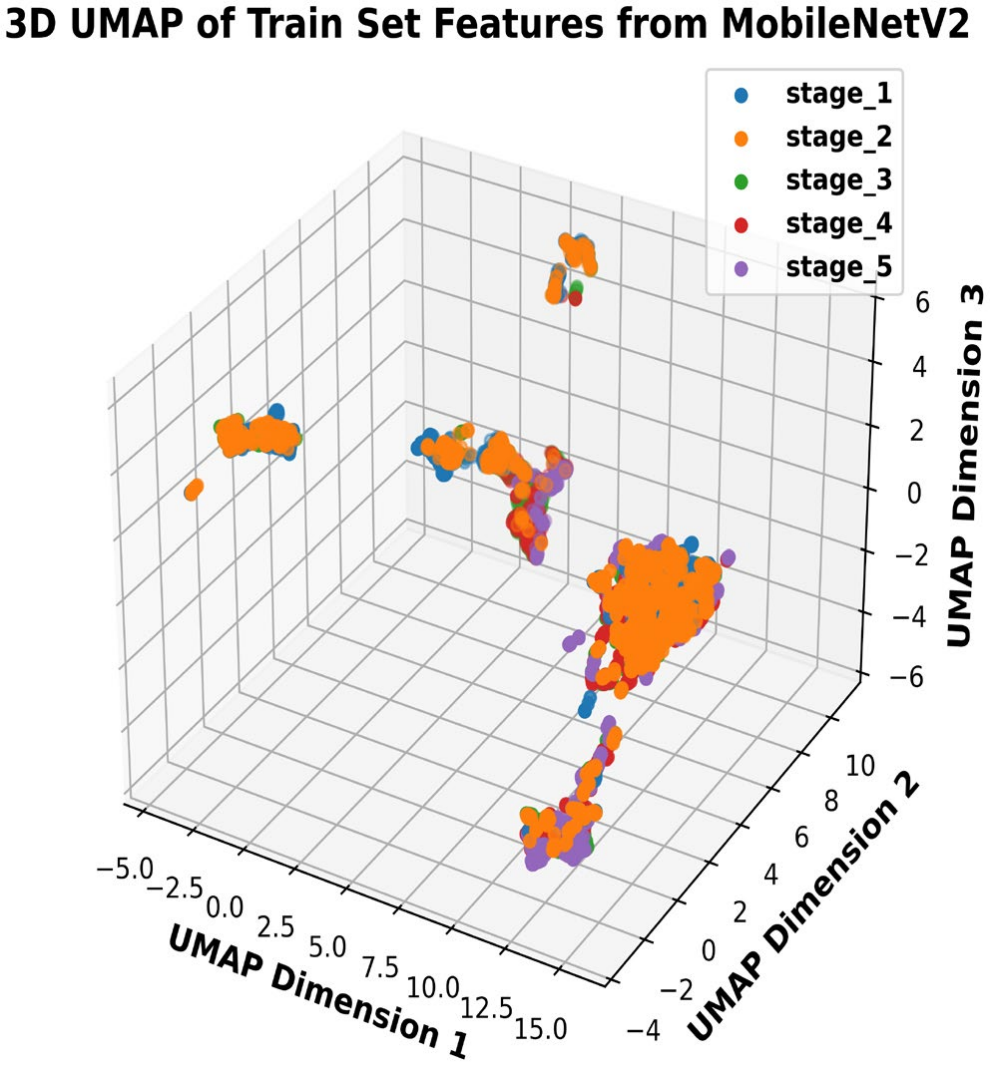
UMAP visualization of feature overlap in raw images.

**Fig. 12:**
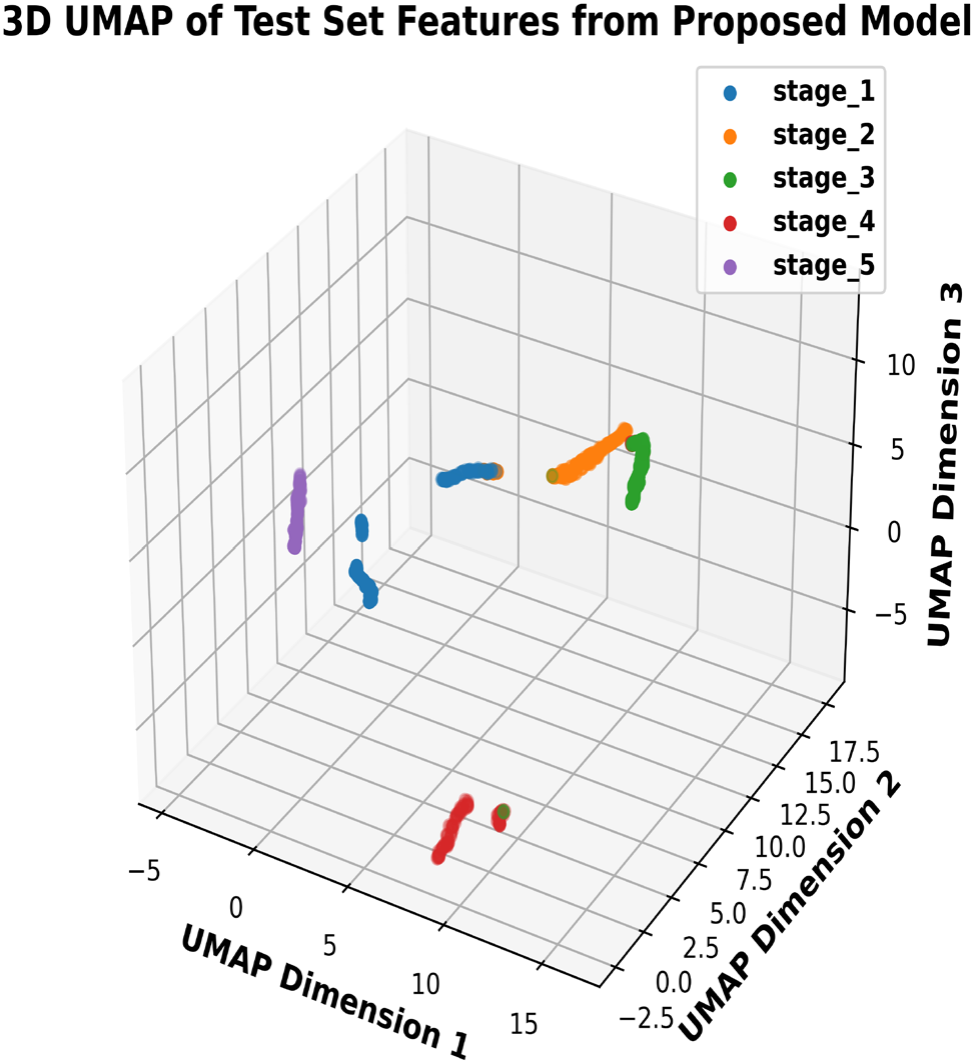
UMAP visualization of features from the proposed model.

To visually analyze feature embeddings, we used UMAP to project the BLBVisionDB dataset. Figure 11 demonstrates significant class-wise overlap in the raw image features, particularly between stages 2, 3, and 4. This overlap highlights the inherent complexity of the dataset and the challenge of distinguishing between classes with severe similarities. Such overlap underscores the need for advanced feature extraction techniques, such as multiclass segmentation or more sophisticated models, to improve stage divisibility and overall performance. In contrast, Fig. 12 illustrates the UMAP visualization of feature embeddings generated by the proposed multitask model. The features are much better separated, with distinct clusters formed for each class. This improved separation demonstrates the model’s effectiveness in learning discriminative features, significantly reducing class overlap and enhancing classification performance. The clear boundaries between clusters validate the robustness of the proposed approach in handling complex datasets such as BLBVisionDB.

**Table 9:** Performance comparison of different Strategies for BLB disease classification.

Method	Accuracy (%)	Precision (%)	Recall (%)	F1-score (%)
Raw Images (Baseline)	89.58	90.24	89.58	89.56
CBAM Attention Module	92.46	93.39	92.53	92.71
Detectron Masks + CBAM	**96.23**	**95**	**95**	**95**

Table 9 provides a detailed comparison of the three evaluation methods. The results demonstrate that the inclusion of the CBAM attention module and the Detectron generated masks effectively enhances the model’s ability to focus on diseased regions, resulting in superior classification performance. The integration of CBAM enabled the model to prioritize relevant features through channel-wise and spatial attention, while the Detectron masks further refined the diseased region localization. The confusion matrices from Fig. 7 and Fig. 9 highlight improvements in classification accuracy across the severity stages. The enhanced method with CBAM and detector masks shows a significant reduction in misclassifications, especially in the intermediate stages (*stage_3* and *stage_4*), where the baseline model previously exhibited a notable confusion.

**Fig. 13:**
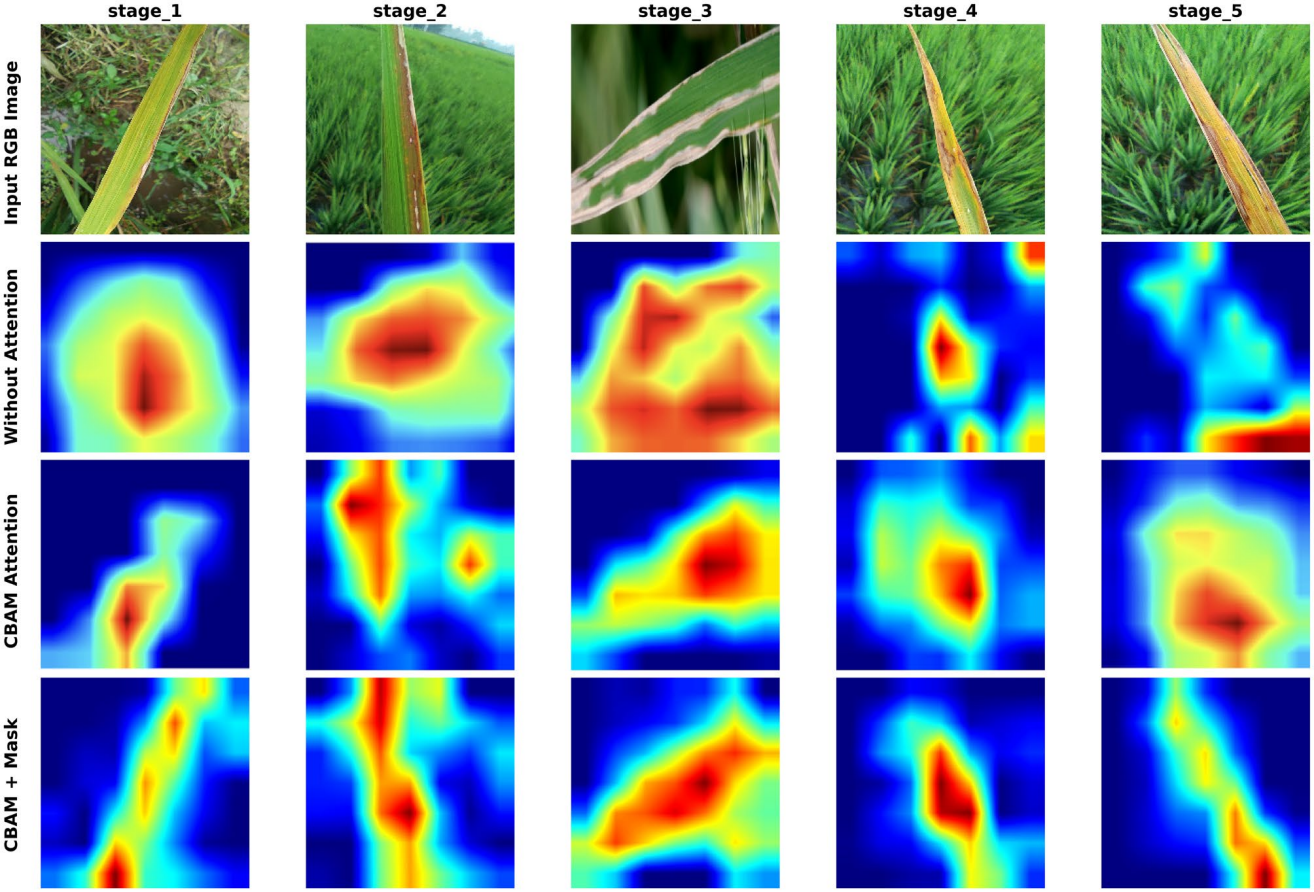
Comparative analysis of Grad-CAM heatmaps for rice BLB classification.

To further evaluate the impact of individual components in the proposed multitask detection model, we analyzed Grad-CAM visualizations from various configurations. Figure 13 presents a comparative analysis of Grad-CAM heatmaps for classifying bacterial leaf blight (BLB) in rice. The first row presents the original RGB images of infected rice leaves. The second row highlights the Grad-CAM outputs from the MobileNetV2 model without any attention mechanism, showing limited focus on disease-relevant areas. In contrast, the third row demonstrates the effectiveness of the CBAM attention model, where the heatmaps show improved localization of critical diseased regions. The final row integrates CBAM attention features with Detectron masks, producing heatmaps that closely align with the actual diseased areas, indicating enhanced model performance in disease localization and classification. This analysis underscores the value of attention mechanisms and mask-assisted models in improving prediction accuracy for bacterial leaf blight (BLB).

### Inference time evaluation and deployment considerations

To evaluate the practical deployment feasibility of the proposed RCAMNet architecture, we measured the average inference time of the complete pipeline, comprising three stages: segmentation using Detectron2, feature enhancement via the CBAM attention module, and severity classification with MobileNetV2. The inference time was measured on a CUDA-enabled NVIDIA Tesla V100 GPU within a Google Colab Pro environment. The average time taken by each stage is summarized in Table 10. The overall end-to-end processing time per image was found to be approximately 206.88 ms, with the segmentation stage being the most computationally intensive. Despite this, the modularity and efficiency of the lightweight MobileNetV2 backbone and CBAM module contribute to maintaining a near real-time performance level. For deployment in field scenarios, we plan to optimize the model using lightweight formats such as TensorFlow Lite and ONNX Runtime, and benchmark them on edge devices including smartphones and embedded GPUs. These adaptations will help reduce latency and power consumption while retaining accuracy.

**Table 10:** Stage-wise average inference time of RCAMNet on NVIDIA Tesla V100 GPU.

Processing Stage	Average Inference Time (ms)
Segmentation (Detectron2)	134.91
CBAM Feature Enhancement + MobileNetV2 Classification	71.97
**Total Pipeline Inference Time**	**206.88**

## Discussion

### Contextualization of results

The results of this study demonstrate the potential of RCAMNet to fill a critical gap in rice BLB severity detection. Currently, there is no established system for the reliable identification of BLB stage, despite the rapid progression of the disease and the severe impact on rice yield. RCAMNet addresses this need by enabling stage-wise severity assessment, which is valuable for timely interventions by farmers and agricultural officers. Additionally, the dataset curated in this work is a significant contribution, as no publicly available dataset previously existed for BLB progression stages. The model also performs robustly under real-world conditions such as occlusion, low light, and overlapping symptoms, highlighting its practicality in diverse agricultural environments. Compared to traditional CNN-based approaches, RCAMNet shows improved accuracy and interpretability.

### Limitations

Despite these strengths, the proposed approach has some limitations. A major constraint is deployment compatibility: the segmentation stage relies on Detectron2, which is currently not suitable for mobile devices. Consequently, users must upload images to a server for segmentation, which introduces latency and connectivity dependence. This limitation could be addressed in future work by developing a lightweight, mobile-compatible multiclass segmentation model. Furthermore, the data set used in this study was collected exclusively from Kerala, India, which may limit the applicability to other regions with different environmental conditions and disease presentations.

### Future work

Future research will focus on developing a compact, mobile-friendly version of RCAMNet with reduced inference time and on-device processing. Incorporating user feedback into classification results, expanding the dataset to include more geographic and environmental variability, and building multilingual, real-time monitoring applications are also planned.

## Conclusion

In this study, we proposed a novel three-phase RCAMNet framework to analyze the progression of Bacterial Leaf Blight (BLB) in rice, addressing critical challenges in plant disease classification. The foundation of this research is the development of a comprehensive dataset, *BLBVisionDB*, which includes 5,896 high-quality images collected from infected paddy fields in Kerala and artificially inoculated plants grown under controlled greenhouse conditions. The dataset was manually categorized into five severity classes based on Leaf Area Infected (LAI), offering a robust resource for analyzing disease progression. Initially, we evaluated transfer-learned Deep Convolutional Neural Network (DCNN) models, specifically MobileNetV2, on raw images. These models faced challenges due to similar disease patterns and noisy backgrounds, achieving an accuracy of 89.58%. To overcome these limitations, we integrated a Convolutional Block Attention Module (CBAM) for feature enhancement, significantly improving the model’s ability to focus on disease-relevant regions. This approach improved classification performance, with MobileNetV2 + CBAM achieving a test accuracy of 92.46%, along with notable gains in precision, recall, and F1 score. To further refine performance, particularly for field-based applications, we incorporated supervised segmentation techniques to generate multiclass segmentation masks using models such as MultiClass U-Net, DeepLabv3, and Detectron2. Detectron2 outperformed other methods with a mean intersection over Union (IoU) score of 59. 49%, and effectively isolating regions of interest (ROI) for enhanced feature extraction. The features from the multiclass segmentation masks were further enhanced using the CBAM model and merged with raw image features. This merged feature approach highlighted core diseased areas, leading to a significant improvement in classification accuracy, achieving a test accuracy of 96.23%. Additionally, Grad-CAM visualizations provided critical insights into the decision-making process, showcasing the model’s ability to focus on disease-specific features such as lesions and yellowing. These visualizations underscore the reliability of the proposed method in accurately identifying and classifying the various BLB severity phases. These advances have immense potential to revolutionize the management of agricultural diseases, promote sustainable farming practices, and improve crop resilience in diverse farming conditions.

## Data Availability

The custom dataset for rice bacterial leaf blight (BLB), named BLBVisionDB, used in this study is hosted at https://sudheshkm.github.io/Bacterial-Leaf-Blight-disease-progression/. The dataset is made available from the corresponding author upon reasonable request.
